# Response to the letter to the editor on the article: radiomics nomogram based on optimal VOI of multi-sequence MRI for predicting microvascular invasion in intrahepatic cholangiocarcinoma

**DOI:** 10.1007/s11547-024-01799-7

**Published:** 2024-03-21

**Authors:** Qing Wang, Xianling Qian, Xijuan Ma, Baoxin Qian, Xin Lu, Yibing Shi

**Affiliations:** 1 Graduate Department, Bengbu Medical College, Bengbu, 233000 Anhui People’s Republic of China; 2 Department of Radiology, Xuzhou Central Hospital, Xuzhou Clinical School of Xuzhou Medical University, No.199 Jiefang South Road, Quanshan District, Xuzhou, 221009 Jiangsu People’s Republic of China; 3 Department of Radiology, Zhongshan Hospital, Fudan University, No.180 Fenglin Rd, Shanghai, 200032 People’s Republic of China; 4 Shanghai Institute of Medical Imaging, No.180 Fenglin Rd, Shanghai, 200032 People’s Republic of China; 5 Department of Cancer Center, Zhongshan Hospital, Fudan University, No.180 Fenglin Rd, Shanghai, 200032 People’s Republic of China; 6 Huiying Medical Technology, Huiying Medical Technology Co., Ltd, Room A206, B2, Dongsheng Science and Technology Park, HaiDian District, Beijing, 100192 People’s Republic of China; 7 Department of Radiology, Shanghai Geriatric Medical Center, No. 2560 Chunshen Rd, Shanghai, 201104 People’s Republic of China

Dear Editor,

We want to express our gratitude to Dr. Xie and colleagues for their feedback on our research [1] and hope that this letter proves helpful to the field of radiomics research.

As Xie et al. pointed out, due to the nature of our study being a retrospective study, it included nine different MRI scanners from four manufacturers. However, the study also reflects the current clinical practice situation. Most MRI radiomics studies, even multicenter studies, have used data sets that were intentionally from a single manufacturer and a single field strength scanning instrument, which do not represent the generalizability desired for AI model [2]. Mes et al. [3] also found that variability in the data appeared to be independent of manufacturer and field strength variations. A study focused on “real world radiomics” analyzed the heterogeneity of different machine-based models and found that the prediction ability of radiomics classification models using multi-manufacturers data is consistent with previous single-manufacturer studies [2].

The comment made by Xie et al. emphasizes the importance of image resample due to potential variations in data acquisition and processing caused by the utilization of different MRI scanners. We also quite agree with this view, which is also a limitation of our study. However, there is currently no specific recommendation for voxel interpolation. The IBSI guidelines [4] also suggest that downsampling may result in information loss while upsampling may introduce non-realistic information due to image layer thickness. Additionally, the order of resampling also needs to be considered: before VOI segmentation or before feature extraction, different sampling orders may affect research results. Currently, there is no specific research comparing different resampling voxel sizes and resampling orders for intrahepatic cholangiocarcinoma in terms of radiomics. Our next research plan is to adopt multi-scaling strategies based on our research, hoping to provide more reference information for the standardization of radiomics research.

**Fig. 1:**
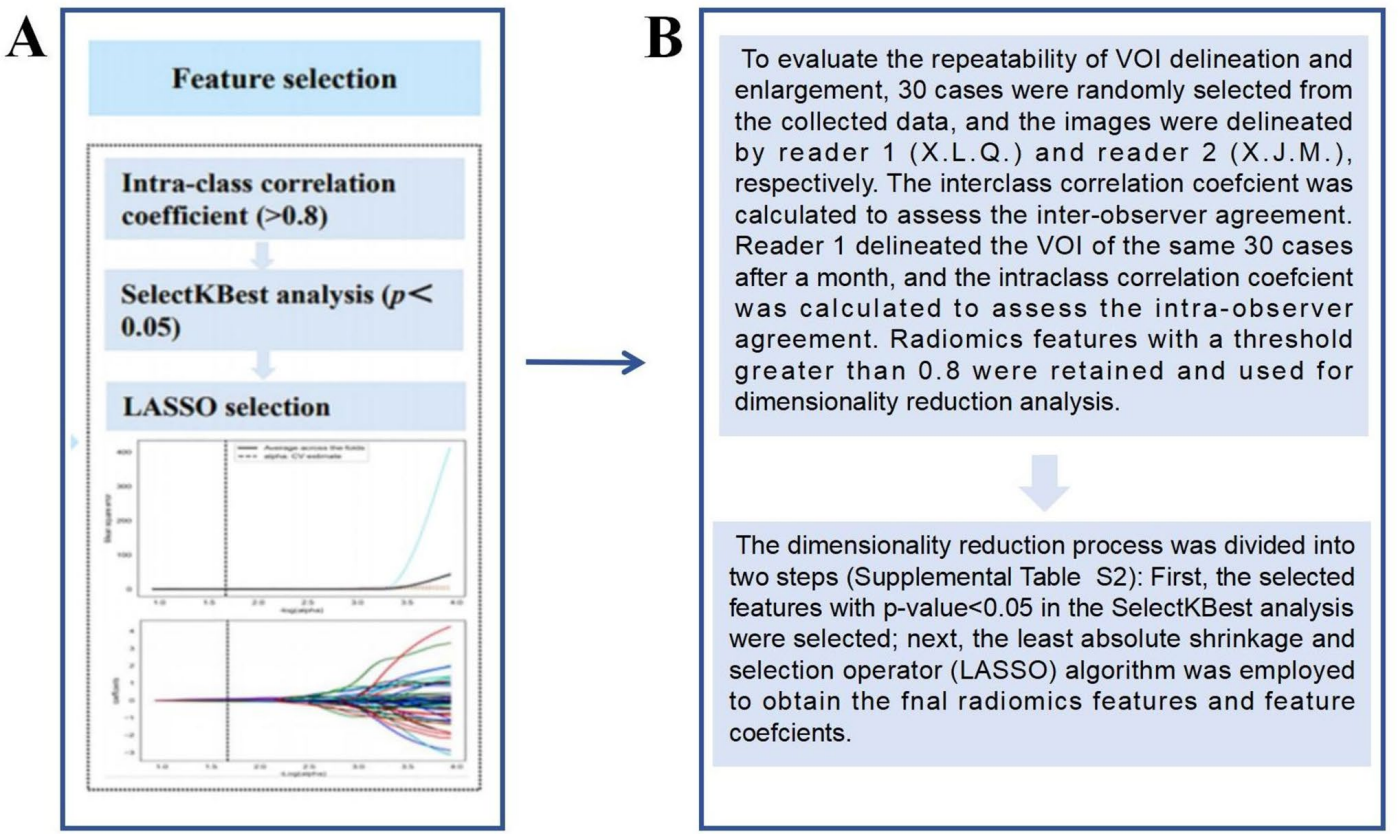
The process of data dimensionality reduction in the main text. **A** Figure 2 of the main text, **B** “Feature extraction and selection” section of the main text

**Fig. 2:**
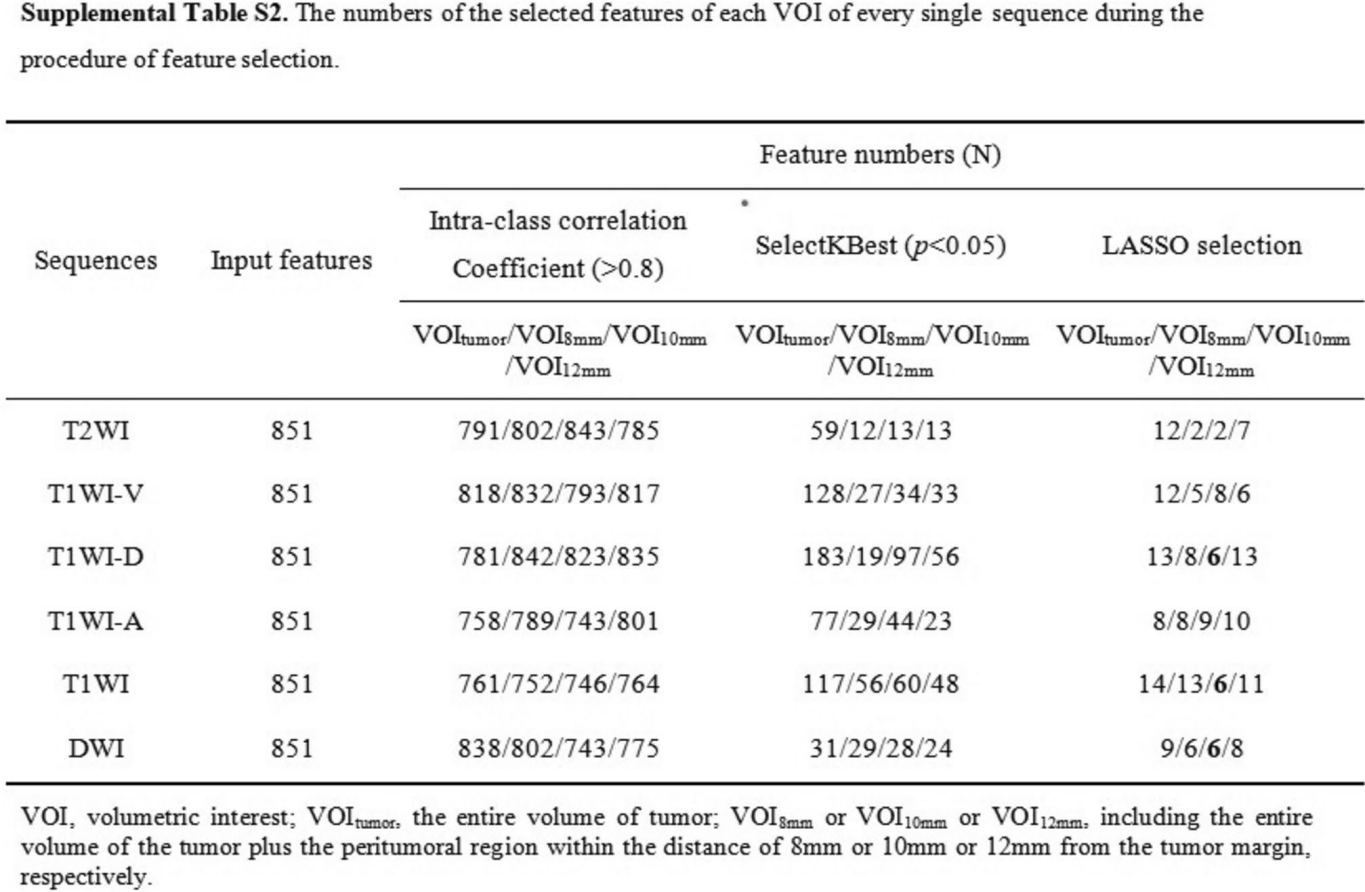
The process of data dimensionality reduction in supplementary materials (Table S2)

We agree with Xie et al.'s comments regarding data dimensionality reduction, and we have also done so in our study. We have explained that in the “Feature extraction and selection” section of the main text: Firstly, the reliability of radiomics features was calculated by using intra-class correlation coefficient. Features with intra-class correlation coefficients > 0.8 in both intra-observer and inter-observer settings were considered as reproducible radiomics features and were chosen for subsequent investigation. Then, we performed SelectKBest and the least absolute shrinkage and selection operator algorithm to ensure feature stability and achieved good performance in the final nomogram. We have demonstrated the process and results of “feature extraction and selection" in Fig. 2 (Fig. 1) of the main text and Table S2 (Figure 2) of the supplementary materials, where the third column of Table S2 represents the intra-observer and inter-observer agreement results. The fourth column represents the results of SelectKBest, and the fifth column represents the least absolute shrinkage and selection operator.

Our novelty lies in the finding that the radiomics model incorporating a 10-mm peri-tumor and intra-tumor area effectively predicts the occurrence of microvascular invasion (MVI). This is consistent with the pathological basis that MVI tends to occur at the tumor margin [5, 6]. The 10-mm result is also consistent with Chong et al. [7] and Feng et al. [8] prediction of MVI in hepatocellular carcinoma.

In conclusion, we appreciate Dr. Xie et al. comments as they are both highly relevant and important questions in current radiomics research. We hope that our response can provide some insight into the research process of radiomics. We will also insist on this aspect of research to make the radiomics model we have established more clinically valuable and practical. Thanks again to Dr. Xie et al. for their letter and valuable professional advice!
